# Molecular Simulations of PEGylated Biomolecules, Liposomes, and Nanoparticles for Drug Delivery Applications

**DOI:** 10.3390/pharmaceutics12060533

**Published:** 2020-06-10

**Authors:** Hwankyu Lee

**Affiliations:** Department of Chemical Engineering, Dankook University, Yongin 16890, Korea; leeh@dankook.ac.kr

**Keywords:** PEGylation, molecular dynamics simulation, drug delivery, protein, peptide, liposome, dendrimer, carbon nanotube

## Abstract

Since the first polyethylene glycol (PEG)ylated protein was approved by the FDA in 1990, PEGylation has been successfully applied to develop drug delivery systems through experiments, but these experimental results are not always easy to interpret at the atomic level because of the limited resolution of experimental techniques. To determine the optimal size, structure, and density of PEG for drug delivery, the structure and dynamics of PEGylated drug carriers need to be understood close to the atomic scale, as can be done using molecular dynamics simulations, assuming that these simulations can be validated by successful comparisons to experiments. Starting with the development of all-atom and coarse-grained PEG models in 1990s, PEGylated drug carriers have been widely simulated. In particular, recent advances in computer performance and simulation methodologies have allowed for molecular simulations of large complexes of PEGylated drug carriers interacting with other molecules such as anticancer drugs, plasma proteins, membranes, and receptors, which makes it possible to interpret experimental observations at a nearly atomistic resolution, as well as help in the rational design of drug delivery systems for applications in nanomedicine. Here, simulation studies on the following PEGylated drug topics will be reviewed: proteins and peptides, liposomes, and nanoparticles such as dendrimers and carbon nanotubes.

## 1. Introduction

**Figure 1:**
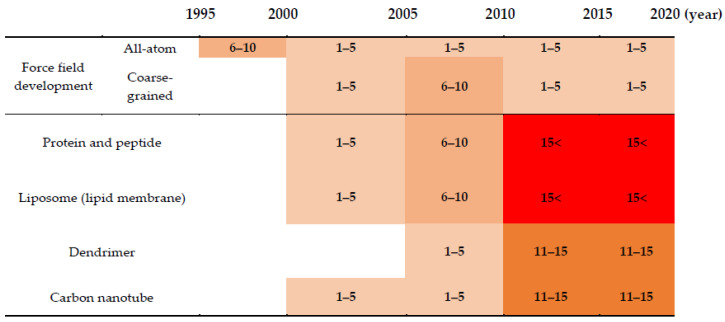
Number of publications on the force-field development and molecular dynamics simulations of polyethylene glycol (PEG)ylated biomolecules and nanoparticles (the numbers of publications, 1–5, 6–10, 11–15, and 15<, are represented in the order of light to dark red colors).

Polyethylene glycol (PEG) and polyethylene oxide (PEO), which are hydrophilic polymers composed of the subunit –(CH_2_–CH_2_–O)*_n_*–, have been often covalently or noncovalently attached to the surfaces of drug molecules or transporters, a process called PEGylation [1,2]. PEG chains can sterically shield the encapsulated drugs from plasma proteins in the bloodstream and maintain good water solubility, leading to increased circulating lifetime and decreased cytotoxicity [3,4,5,6,7,8,9]. Therefore, the PEGylation method has been widely applied to pharmaceutical industries over the past three decades, since the first PEGylated protein was clinically approved by the FDA in the early 1990s [10,11]. Despite this successful application of PEGylation, the effects of PEG size, structure, and grafting density on the efficiency of drug delivery have not been well interpreted because of the limited resolution of experimental techniques. To complement experimental observations at nearly the atomic scale, molecular dynamics (MD) simulations have been performed. Figure 1 lists the number of publications on the development of PEG force fields (FFs) and MD simulations of PEGylated biomolecules and nanoparticles. Since all-atom and coarse-grained (CG) PEG FFs have been developed after 1995, short linear PEG chains were mainly simulated up until the 2000s, but recent advances in computer power and simulation methodologies have allowed many more simulations of large complexed (e.g., branched) PEG chains, PEGylated nanoparticle (or protein)-drug complexes and their self-assemblies interacting with plasma proteins and lipid membranes [12,13,14]. In particular, simulations have revealed that PEGylation influences the conformations and surface properties of drug molecules or transporters and thus modulates the efficiency of drug delivery and release, which helps explain atomic-level phenomena and determine the optimal size, structure, and density of PEG for drug delivery applications [12,14]. Note that the conformation of PEGs and their interactions with other molecules can be reasonably predicted only in the presence of accurate FFs. For the development of all-atom [15,16,17,18,19,20,21,22,23] and CG models [24,25,26,27,28,29,30,31] for PEG and PEO, previous review papers are recommended [12,13,14]. 

In this review, we will first (Section 2) review MD simulations of PEGylated biomolecules such as proteins, antimicrobial peptides (AMPs), and coiled coil peptides, focusing on their structural changes induced by PEGylation. Next (Section 3), MD simulations of PEGylated liposomes interacting with drugs and plasma proteins will be reviewed, which are interpreted by the polymer theory regarding the effect of PEG size and density on protein adsorption. Lastly (Section 4), we will focus on simulations of PEGylated nanoparticles such as dendrimers and carbon nanotubes (CNTs).

## 2. PEGylated Biomolecules: Proteins, Antimicrobial Peptides, and Coiled Coil Peptides

Since Abuchowski et al. found the effect of PEGylation on immunogenicity and circulating lifetime of serum albumin proteins in 1977 [32,33], PEGylated protein-based drugs or drug transporters have been clinically approved such as Neulasta [34], Oncaspar [35], Cimzia [36], Mircera [37], Omontys [38], Macugen [39], Plegridy [40], Krystexxa [41], Adagen, Pegasys, Sylatron, PEGlntron, Somavert [3,10,42,43]. Although PEGylation has been successfully applied for protein- and peptide-based pharmaceutics [7,44,45,46], the structural stability and surface properties of PEGylated proteins need to be understood to increase drug delivery efficiency. Since the structural change of proteins cannot be negligible, most simulation studies have been performed using all-atom models rather than CG models, although some CG simulations have shown the conformation of the grafted PEG. Here, PEGylated proteins, AMPs, and coiled coil peptides will be reviewed.

### 2.1. Proteins

Manjula et al. performed all-atom simulations of PEGylated hemoglobin and showed that PEG chains on the protein surface have the folded structure rather than the extended conformation, which weakens the interactions between hemoglobin and other biopolymers [47,48]. The Liu group simulated PEGylated insulin in water, showing that PEG chains do not only interact with hydrophobic residues of insulin but also form hydrogen bonds with water, leading to the increased size and stability of insulin, which helps explain the longer circulating lifetime as observed in experiments [49]. They also investigated the interactions of PEG with lysozyme [50] and cytochrome c [51], showing the effect of PEGylation on the conformation and stability of proteins at different temperatures. Xue et al. simulated a series of five small peptides grafted with PEG, showing that PEGylation more significantly influences the conformations of charged peptides than those of neutral peptides [52]. Mu et al. showed that PEG size and PEG-conjugated position modulate the hydrodynamic volume of the Staphylokinase–PEG complex and the flexibility of grafted PEG chains [53]. Khameneh et al. simulated PEGylated human growth hormone with its receptor and performed the docking analysis, showing the random-coil formation of PEG and the reduced binding affinity between human growth hormone and its receptor in the presence of PEG [54]. The Accardo group simulated PEGylated hexa-phenylalanine [55,56], tetra-tryptophan [57], and tyrosine-containing aromatic peptides [58], showing the effect of PEGylation on the conformation and stability of assembled structures. 

**Figure 2:**
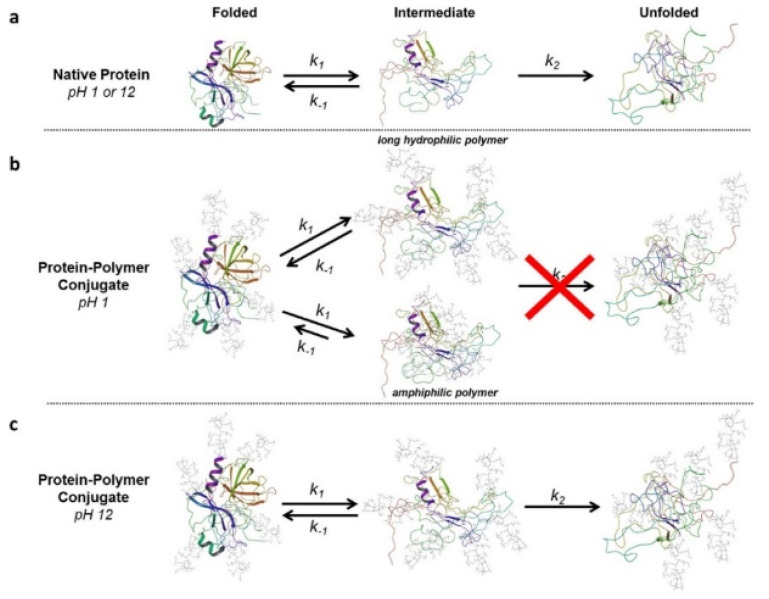
Unfolding pathways at different pH for (**a**) native chymotrypsin at pH 1 and 12, (**b**) PEGylated chymotrypsin at pH 1, and (**c**) PEGylated chymotrypsin at pH 12. PEG chains stabilize partially unfolded intermediate states and thus inhibit irreversible denaturation at pH 1 but not at pH 12 (reprinted with permission from [67]. Copyright (2018) American Chemical Society).

Recently, the interactions between PEG and individual amino acids have been quantified and applied to predict the extent of protein folding. Settanni et al. simulated a mixture of PEG and plasma proteins such as serum albumin, transferrin, complement Cq1, and apolipoprotein A1, and calculated the local density of PEG near individual amino acids, the preferential binding coefficient of each peptide for PEG [59,60], and the conformation and thickness of PEG layer adsorbed on the protein surface [61], showing that PEG–protein interactions can be quantified by a simple model in terms of the solvent-accessible surface area exposed by each amino acid type on the protein surface, favorably compared with experimental results obtained by label-free proteomic mass spectrometry. Kurinomaru et al. [62], Zaghmi et al. [63], and Sindhu et al. [64] found the effect of PEGylation on the structure, dynamics, and binding affinity of enzyme-therapeutic drugs such as α-amylase, glutamate dehydrogenase, and L-asparaginase, respectively. The Colina group reparameterized non-bonded potential parameters of the MARTINI CG PEG FF that was previously developed by Lee et al. [25] and Rossi et al. [26], which allows the accurate prediction of the interactions between PEG and proteins [65]. Using this CG model, they simulated PEG interacting with plasma proteins such as bovine serum albumin, human serum albumin, and apo-human serum transferrin, which reasonably predict the experimentally observed local densities of PEG around individual amino acids [65,66]. In particular, they simulated PEGylated chymotrypsin (a digestive enzyme), showing that PEG chains stabilize partially unfolded intermediates and even help the refolding to an active conformation, to an extent dependent on pH as described in Figure 2 [67], which supports the experimental hypothesis regarding the effect of PEG on protein folding and helps in the rational design of protein–polymer conjugates. They also observed the dependence of the PEG–peptide hydrogel interaction on peptide sequence and solvent condition [68].

### 2.2. Antimicrobial Peptides

AMPs are cationic amphipathic peptides composed of less than 50 amino acids that can be extracted from eukaryotic organisms for their defense mechanism [69]. Cationic AMPs selectively bind to anionic bacterial cell membranes rather than neutral human cell membranes and thus have been considered a promising possible antibiotics [70,71]. To achieve this, the high concentration of AMP is required, but AMP at high concentrations can nonspecifically attack human cells, leading to a decrease in the efficiency of specific targeting, which limits the application of AMP as antibiotics. To overcome this, PEGylation has been experimentally applied to AMPs such as nisin [72], magainin 2, tachyplesin I [73,74], KYE_2_8 [75], LL-37 [76] and synthetic AMPs (CaLL [77] and M33 [78]), showing decreased antimicrobial activity and increased solubility, which has motivated simulation studies on the interactions between AMP and PEG.

**Figure 3:**
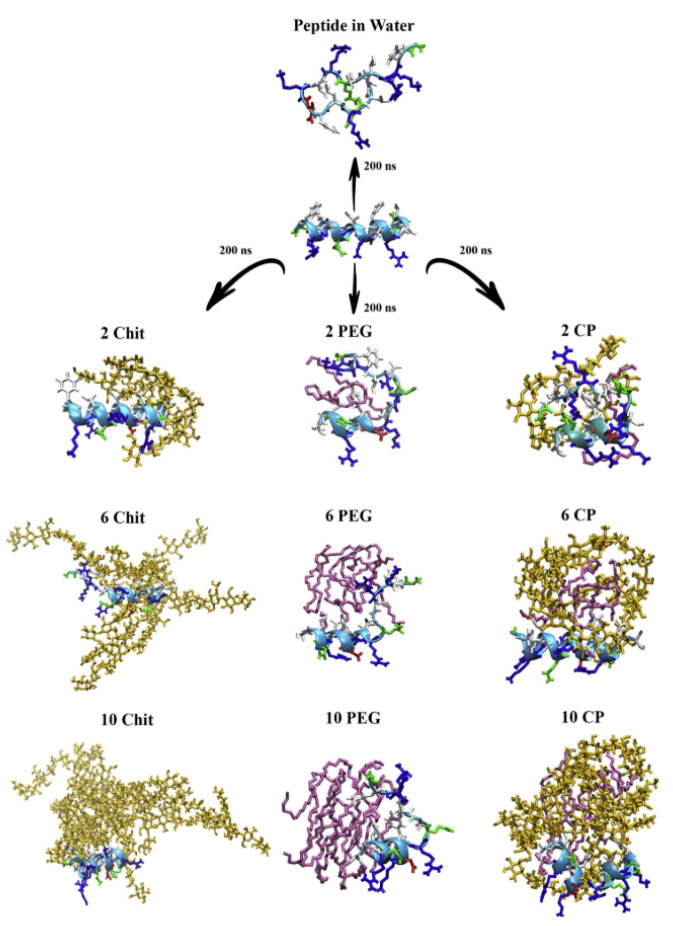
Snapshots of antimicrobial peptide GF-17 interacting with chitosan (**left**), PEG (**middle**), and both chitosan and PEG (**right**) (reprinted with permission from [84]. Copyright (2020) Elsevier).

Wu et al. performed all-atom simulations of cecropin P1 grafted to the silica surface via a PEG cross-linker, showing the effect of PEG size and ionic strength on the conformation and antimicrobial activity of the peptide [79,80]. Our group simulated PEGylated magainin 2 and tachyplesin I interacting with lipid bilayers, showing that PEGylation reduces the binding strength between peptides and bilayer surfaces, which occurs more significantly for α-helical magainin 2 than for β-sheet tachyplesin I [81]. Recently, Jafari et al. simulated PEG-encapsulated magainin 2 and found that the PEG–peptide interaction is significantly modulated by aromatic and basic residues of the peptide [82], and Souza et al. simulated the insertion of PEG-encapsulated human beta-defensin-3 to lung surfactant models, showing that PEG chains promote the translocation of the peptide from gas phase to water phase [83]. Asadzadeh et al. simulated GF-17 (17th–32nd residues of LL-37) interacting with chitosan, PEG, or both, showing that the peptide interacts more tightly with PEG than with chitosan (Figure 3), leading to lower helicity in the presence of PEG [84].

### 2.3. Coiled Coil Peptides

Coiled coils are peptides composed of two or more α-helices wound into a superhelix. Sequences of coiled coils contain a heptad repeat of seven amino acid residues, where the 1st and 4th residues of each heptad repeat are hydrophobic [85]. These hydrophobic residues are located in the core of coiled coils and thus stabilize the superhelical structure of coiled coil bundles [85]. Coiled coils are found in approximately 10% of all proteins and serve critical roles as mediators of oligomerization of many proteins such as transcription factors, molecular motors, receptors and signaling molecules [86]. In addition, coiled coils can self-assemble to mechanically rigid protein fibers and thus have been synthesized for drug delivery applications as templates to promote the assembly of other molecules [87,88]. To increase their solubility and stability, PEGylated coiled coils have been experimentally synthesized by the Klok group [89,90,91,92,93], the Kros group [94,95,96,97,98,99,100,101,102,103], and the Xu group [104,105,106,107,108,109,110,111,112,113], showing membrane fusion and micelle assembly modulated by peptide sequence, PEG size and density, which have been theoretically complemented by simulations. 

**Figure 4:**
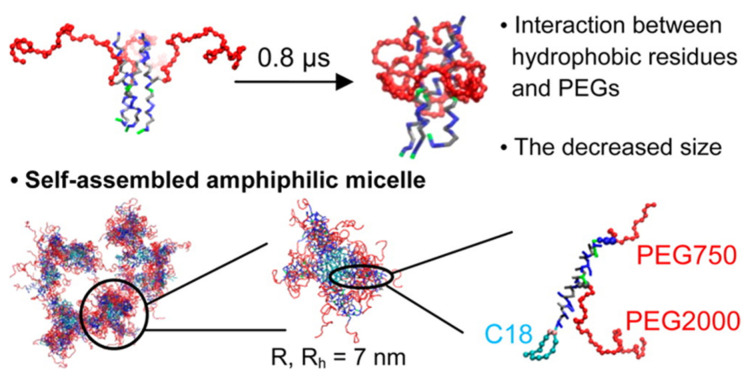
Initial and final snapshots of PEGylated trimeric coiled coils, showing the compact conformation of PEG due to the hydrophobic interaction (top). Snapshots of self-assembled micelles having a hydrodynamic radius of 7 nm for each micelle (bottom) (reprinted with permission from [120]. Copyright (2014) American Chemical Society).

Jain and Ashbaugh performed replica exchange simulations of PEGylated coiled coils, showing the higher helicity of coiled coils grafted with larger PEG chains due to the interactions between PEG and lysine residues of peptides [114]. The Keten group found strong interactions of PEG chains with both hydrophobic and polar residues of peptides, leading to increased helicity and decreased solvent-accessible surface area of the peptide in the presence of PEG, to an extent dependent on solvent hydrophobicity [115]. The binding energy of a cyclic peptide dimer was influenced by PEG length and grafting density [116]. They also found the high helicity and stability of coiled coils grafted with low-molecular weight PEG chains [117], and the extended structure of PEG chains grafted to helix micelles [110]. In particular, PEGylation influences the micelle size and stability, which is interpreted by a competition between the entropy of PEG conformations in the assembled state [111,118]. Recently, they performed both all-atom and CG simulations of PEGylated coiled coils composed of three or four helices, showing the formation of self-assembled micelles and the effect of the coiled coil oligomeric state on micelle size and stability [119]. Our group also performed all-atom and CG simulations of PEGylated trimeric coiled coils and their self-assembled micelles (Figure 4), and calculated their radii of gyration and hydrodynamic radii, which favorably compare with experimental values [120]. In particular, we found that hydrophobic residues in the exterior sites of coiled coils tend to be less exposed to water and thus interact with PEG, leading to the compact conformation of adsorbed PEG [120].

## 3. PEGylated Liposomes

Liposomes, which are synthetic vesicles composed of phospholipid membranes, can transport drug molecules across specific cell membranes and thus have been widely used for drug delivery applications [121,122,123]. To increase solubility and circulating time of drug-encapsulating liposomes, PEG has been often attached to the liposome surface [2], since the first PEGylated liposomal doxorubicin (hydrophobic anticancer drug) was approved by the FDA in 1995 [124]. As the PEG size and concentration increase, encapsulated drug molecules can be more safely shielded from plasma proteins in the bloodstream, but also liposome membranes become unstable [125,126]. Therefore, many experiments have been performed to determine the maximum size and grafting density of PEG that can still maintain liposome stability [125,127,128,129,130,131,132,133,134,135,136,137,138,139,140], which has motivated theoretical studies on the effect of PEG size and grafting density on the conformation and dynamics of PEG chains grafted on the surface.

**Figure 5:**
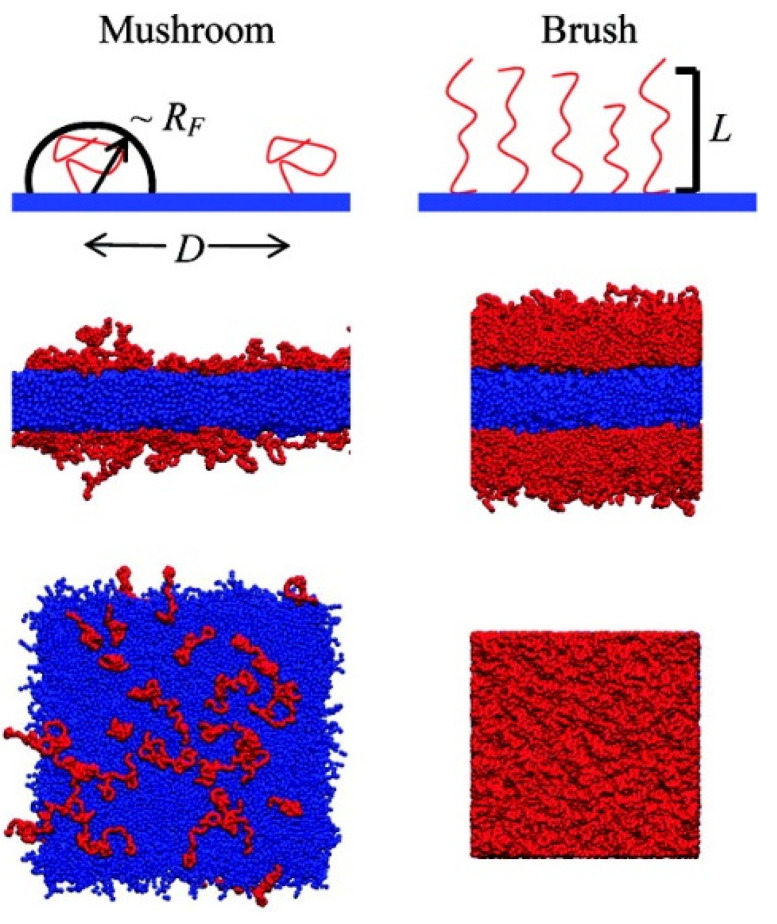
Schematic illustrations of the mushroom and brush conformations (**top**), and snapshots of the side (**middle**) and top-down (**bottom**) views at the end of simulations of PEG chains grafted on a hydrophobic surface (reprinted with permission from [25]. Copyright (2009) American Chemical Society).

The Alexander–de Gennes theory has been applied to predict the transition of hydrophilic polymer chains between hemisphere (mushroom) and brush-like states on the surface [141]. Briefly, at very low grafting density, the grafted chain behaves like an isolated chain in solution, leading to a hemisphere (mushroom) conformation with a size given by the Flory radius, *R*_F_ = *aN*^3/5^, where *N* is the degree of polymerization and *a* is the monomer size (Figure 5). At high grafting density (*D < R*_F_), polymer chains become crowded and repel each other, leading to a brush-like conformation with a thickness given by *L* = *Na*(*a*/*D*)^2/3^, where *D* is the distance between the grafting points of polymers. Jeon et al. calculated free energies of steric repulsion, van der Waals attraction, and hydrophobic interaction for the binding between spherical model proteins and PEO chains grafted on the hydrophobic surface, to an extent dependent on PEO length and grafting density [142]. Their free-energy calculations show that longer size and higher density (i.e., the brush state) lead to the optimal protein resistance, although surface density is more influential than chain length [142]. In particular, they determined optimal grafting densities of PEO for differently sized proteins, which was interpreted by steric repulsion and hydrophobic interaction between protein and PEO layer [143]. Szleifer also calculated free energies and showed the dependence of protein adsorption on the PEG density as well as on the protein conformation and concentration [144]. Halperin found that adsorption of small proteins can be repressed by increasing the grafting density, while adsorption of large proteins can be suppressed by increasing the brush thickness [145]. They also distinguish specific and nonspecific attractive interactions between various plasma proteins and PEG brushes [146]. Taylor and Jones found that the amount of proteins adsorbed onto PEGylated gold surfaces exponentially decreases as the brush density increases [147].

**Figure 6:**
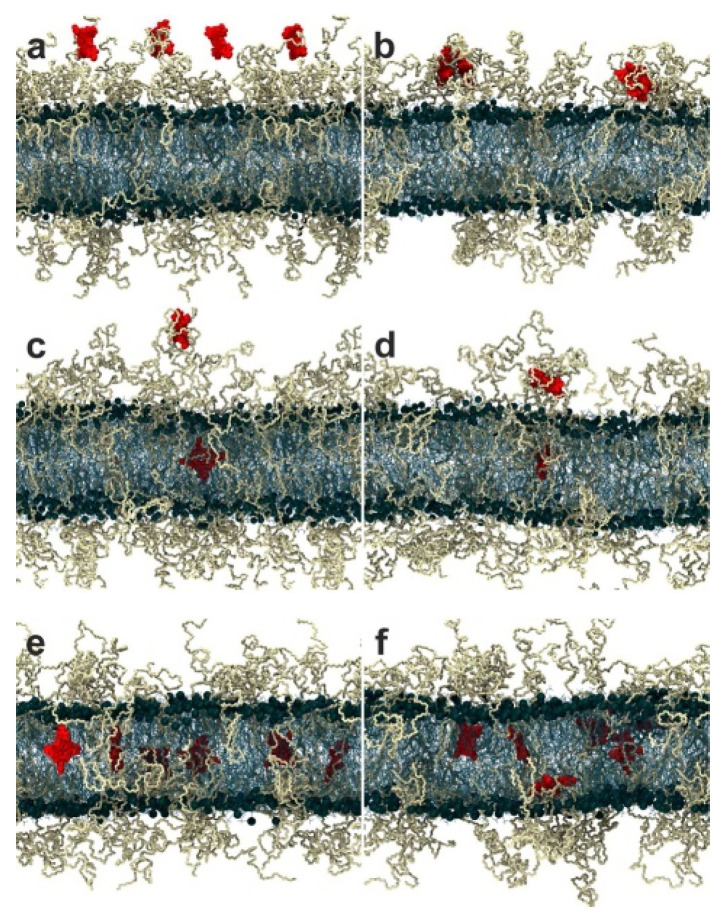
Snapshots of simulations showing the penetration of hydrophobic porphyrins into the PEGylated lipid bilayer as a function of time: four porphyrins at 0 ns (**a**) and 100 ns (**b**); two porphyrins at 0 ns (**c**) and 350 ns (**d**); six porphyrins at 0 ns (**e**) and 350 ns (**f**) (reprinted with permission from [154]. Copyright (2015) American Chemical Society).

To complement these theoretical models, MD simulations have been performed. The Roccatano group performed all-atom simulations of PEGylated lipid bilayers, and their free-energy calculations showed the strong interactions between PEG and lipid headgroups of bilayers [148,149]. Bunker and coworkers parameterized the all-atom PEG model and simulated PEGylated lipid bilayers, showing the interactions between PEG oxygens and Na^+^ ions, and the penetration of PEG chains into a liquid-crystalline membrane but not into a gel-phased membrane [16]. They also found that the strength of the interaction between PEG and salt is modulated by PEG density, salt concentration and type such as NaCl, KCl, and CaCl_2_ [150]. They simulated small peptides interacting with PEGylated lipid bilayers, showing the dependence of peptide penetration on hydrophobicity [151]. In particular, Na^+^ ions bind to lipid bilayers and PEG chains grafted to drug molecules, which induces electrostatic repulsive interactions between lipid bilayers and PEGylated drugs [152]. PEGylation modulates the effect of cholesterol on the conformation and dynamics of lipid bilayers [153]. Their simulations also captured the insertion of hydrophobic drug or light-sensitizing molecules (e.g., porphyrins, indocyanine green, itraconazole, and piroxicam) to the PEG layer and the hydrophobic region of the bilayer (Figure 6) [154,155,156,157,158]. Recently, they simulated linear and branched PEG chains grafted on lipid bilayers, showing that the architecture and length of PEG–lipid conjugates influence the structure and dynamics of membranes, in agreement with experimental results [159].

Although all-atom simulations have captured the conformation and dynamics of PEGylated bilayers and their interactions with hydrophobic drug molecules and salt ions, the effects of PEG size and grafting density on liposome formation and protein adsorption have not been systematically simulated due to computational limitations of system size and time scale. To resolve this, the Klein group parameterized the CG model for PEG and PEGylated surfactants [160] and investigated the interactions between PEGylated surfactants and lipid bilayers [161] and the conformation of self-assembled PEGylated bicelles [162]. Our group also developed CG PEG model within the framework of the MARTINI FF [163,164], which lumps a monomer of PEG (–(CH_2_–CH_2_–O)*_n_*–; three heavy atoms) into each CG bead [18,25]. This CG PEG model was further parameterized to increase dihedral stability by Rossi et al., showing the effect of PEGylation on the curvature of the surfactant bilayer [26]. Using this CG PEG model, Yang and Faller found that the presence of PEGylated lipid promotes the conformational transition from bilayers to micelles [165]. Hezaveh et al. developed another version of the MARTINI-based PEG model and showed the insertion of block copolymers into lipid bilayers, although their model does not include a dihedral potential and thus cannot reproduce the experimentally observed conformation of PEG chains in water [166].

**Figure 7:**
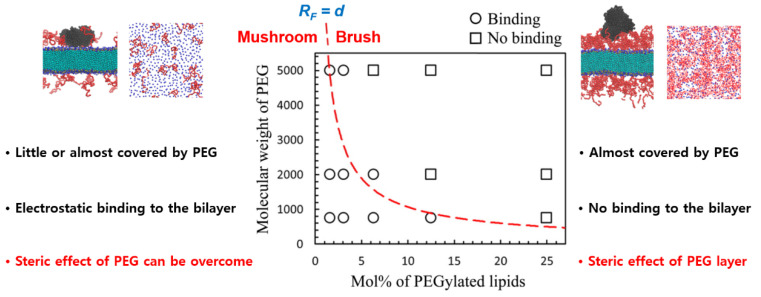
Characterization of binding (circles) and no-binding (squares) between plasma protein (human serum albumin) and the surface of PEGylated bilayer as functions of PEG size and grafting density. The boundary between mushroom and brush states is represented as a thick red line, where the Flory radius equals the distance between the grafting points of PEG (reprinted with permission from [168]. Copyright (2016) American Chemical Society).

Our group simulated a mixture of lipids and PEGylated lipids at different molar ratios, showing the formation of self-assembled liposomes, bicelles, and micelles, respectively, at 0–2.2, 10.5–27.4, and higher mol% of PEGylated lipid, in qualitative agreement with experiments [167]. This indicates that the phase behavior and size of lipid assemblies can be modulated by PEG density because their bulky headgroups increase membrane curvature [167]. Moreover, our CG simulations of PEG chains grafted to a nonadsorbing surface captured the conformational transition between brush and mushroom states, showing good agreement of simulation and Alexander–de Gennes theory [25]. In particular, we characterized the extent of protein adsorption to PEGylated lipid bilayers in terms of different PEG sizes (M_w_ = 750, 2000, and 5000) and grafting densities (1.6–25 mol%), showing that the binding between proteins and membranes is suppressed by the PEG layer in a brush but not in a mushroom (Figure 7), in quantitative agreement with the Alexander–de Gennes theory and experiments regarding much less adsorption of plasma proteins onto the membrane surface grafted with PEG in the brush state than in the mushroom state [168]. It is worth noting that the binding between plasma protein and bilayer surface can be predicted from the boundary between mushroom and brush states of PEG with different sizes and grafting densities, as highlighted in Figure 7. Recently, Sammalkorpi and coworkers showed the formation of self-assembled liposomes, bicelles, and micelles at different PEGylated-lipid concentrations [169,170], similar to our previous work [167].

## 4. PEGylated Nanoparticles

### 4.1. Dendrimers

Dendrimers, which consist of regularly branched monomeric building blocks with many surface terminal groups, have shown great potential for drug delivery applications because of their controlled mass, surface valency, and surface functionality [171]. Drug molecules can be either conjugated to the terminal group of dendrimer or encapsulated into the inner vacancy of dendrimer and then delivered to the desired site [172]. However, charged dendrimers have nonspecific interactions with cell membranes and thus have been neutralized by acetylating their surface terminals. In addition, PEG chains have been often attached to the dendrimer surface, which does not only decrease nonspecific cytotoxicity but also increases dendrimer solubility [173]. In particular, PEG can sterically shield drug molecules from plasma proteins in the bloodstream and thus increase their circulation lifetime [174], which has motivated many simulation studies on the conformation of PEGylated dendrimers and their interaction with drugs, proteins, and lipid membranes.

Tanis and Karatasos performed all-atom simulations of a dendrimer grafted with a single PEO chain, showing the effect of pH on the conformation of PEO and its hydrogen-bond interaction with dendrimer [175]. Karatosas also simulated the complex of PEGylated hyperbranched polyesters and doxorubicin, showing the effects of PEG size and doxorubicin charge on the hydrogen-bond interactions between PEGylated polyesters and doxorubicin [176]. Our CG simulations showed that PEGylation induces interparticle dispersion [177] and the lower extent of cytotoxicity and membrane permeability than acetylation does [178]. In particular, we found that longer chains with higher grafting densities promote PEG–PEG crowding and thus stretch dendrimer terminals towards water, leading to a larger dendrimer with a dense-shell structure [179]. Albertazzi et al. simulated dendrimers containing 2- and 4-arm PEG cores, showing more swollen conformation of dendrimer at higher concentrations of PEG core [180]. Their metadynamics simulations also showed that PEGylated dendrimers have a tight globular shape rather than an open conformation [181]. Pearson et al. showed conformational changes of PEGylated dendrimers at different charge densities [182], and Lin et al. found that PEGylated dendrimers adsorb to lipid monolayers but do not significantly influence the structure and properties of monolayers [183]. 

**Figure 8:**
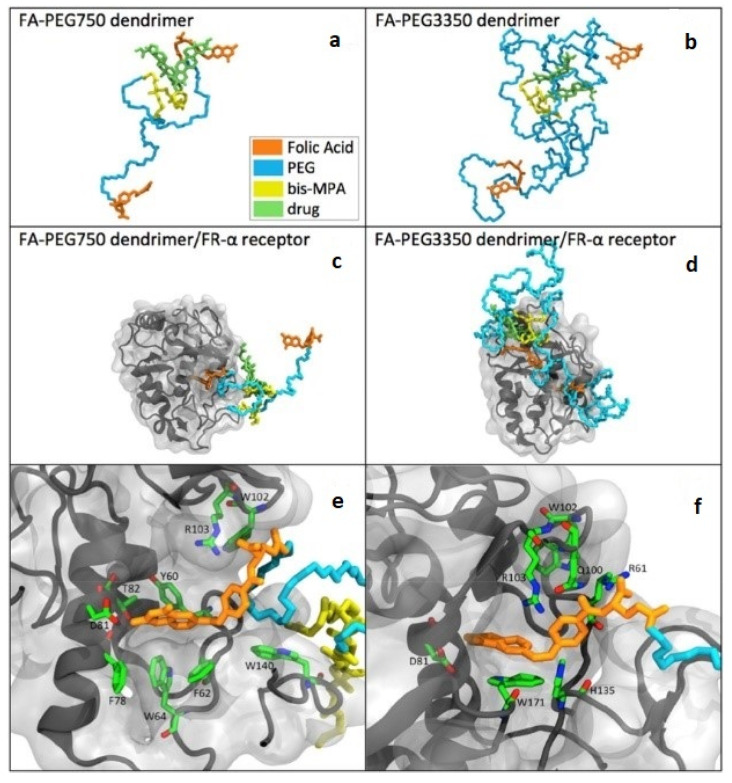
Snapshots of dendrimers complexed with drug, folic acid, and PEG ((**a**) PEG750 and (**b**) PEG3350), and those interacting with a folate receptor (**c**–**f**). (Reprinted with permission from [186]. Copyright (2017) Elsevier).

Recently, large complexes of PEGylated dendrimers and proteins (or drugs) have been simulated. Lim et al. [184] and Barraza et al. [185] respectively simulated paclitaxel and methotrexate drugs interacting PEGylated dendrimers, showing that PEG–PEG crowding decreases the extent of drug release, which helps determine the size and density of PEG for optimal drug release. Sampogna-Mireles et al. simulated dendrimers grafted with PEG and folic acid, showing that PEG chains do not reduce the binding affinity between folic acid and folate receptor (Figure 8) [186], which should be highlighted because their simulations captured the effect of PEG on the binding affinity to the receptor protein. Hsu et al. simulated PEGylated dendron micelle and serum albumin, showing that the penetration of serum albumin into the micelle core can be suppressed by PEGylation [187]. Diaz et al. compared the conformations of dendrimers grafted with PEG or folic acid, showing different effects of PEG and folic acid on dendrimer size, which helps explain the experimentally observed relationship between dendrimer size and circulation time [188]. Wang et al. found that PEGylation significantly weakens the binding between dendrimers and plasma proteins such as human serum albumin and immunoglobulin [189]. Overall, the conformation of PEG and its effect on the internal structure of dendrimer were mainly studied until early 2010s, while the effect of PEGylation on the binding affinity to proteins and drug release efficiency have been more focused for the past five years.

### 4.2. Carbon Nanotubes

Since CNTs are mechanically strong and chemically stable, they have been considered to be good candidate nanomaterials for use as drug transporters [190,191,192]. However, CNTs are highly hydrophobic and thus immediately aggregate in aqueous environment, which limits the application of CNTs at the physiological condition. To overcome this, PEG chains have been covalently or noncovalently attached to the CNT surface. Experiments have revealed the conformation and interparticle dispersion of PEGylated CNTs and their interactions with membranes, proteins, and drug molecules [193,194,195,196,197,198,199,200], which has motivated simulation studies.

In the early 2010s, most simulation studies focused on the conformation of PEG chains and their interactions with the CNT surface. Uddin et al. performed all-atom simulations of CNTs with a mixture of PEO and water, and their free-energy calculations showed the weak adsorption of PEO onto the CNT surface, which were explained by entropic and enthalpic contributions [201]. Our group simulated random adsorption of PEGylated lipids onto the CNT surface [202] and found the effects of PEG size and grafting density on the conformation of PEG grafted onto CNT [203], which favorably compares with the transition of mushroom and brush states in the Alexander–de Gennes theory [141]. Di Crescenzo et al. simulated CNTs interacting with PEG (homopolymer) or PEG–propylene sulfide (PPS) block copolymers and found the stronger interaction of CNT with PEG–PPS than with PEG and the parallel arrangement PEG chains along the tube axis [204]. Aslan et al. compares density profiles of PEGylated lipids adsorbed onto isolated and bundled CNTs, which helps explain their different extents of antimicrobial activity [205]. Sarukhanyan et al. [206] and Han et al. [207] simulated CNTs interacting with various polymers, showing the effect of polymer hydrophobicity on the CNT-polymer conformation and interparticle dispersion. Maatta et al. found the dependence of CNT dispersion on PEG length and CNT diameter [208]. 

**Figure 9:**
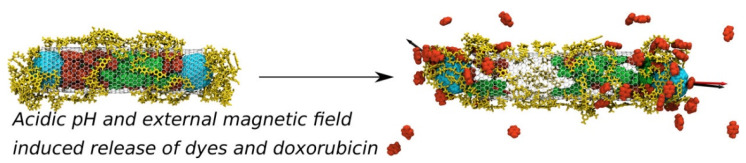
Snapshots of simulations of the carbon nanotubes (CNT) functionalized with PEGylated folic acid (yellow). Doxorubicin (green) and fullerene (light blue) were initially located in the inner cavity of CNT. After 1.5 ns of simulations, doxorubicin molecules are released from CNT at acidic pH but not at neutral pH (reprinted with permission from [217]. Copyright (2018) American Chemical Society).

Recently, large complexes of PEGylated CNTs and other molecules such as lipid membranes, plasma proteins, and anticancer drug molecules have been simulated using all-atom and CG models. Skandani and Al-Haik showed slower penetration of PEGylated CNT into the lipid bilayer than unPEGylated CNT, which was explained by lower adhesion energy of PEGylated CNT [209], as observed in their previous experiments [210]. Our group showed interparticle dispersion and membrane curvature induced by PEGylated CNT [211], and the effects of protein shape, PEG size and grafting density on the adsorption of proteins onto PEGylated CNT [212]. Lin et al. investigated the binding affinity between CNTs and PEGylated proteins such as hormones, neurotransmitter, and vitamin [213], and Hashemzadeh and Raissi showed the adsorption of paclitaxel onto the PEGylated CNT [214]. Kavyani et al. compared the binding strength of CNTs with PEGylated and unPEGylated dendrimers, showing the stronger interactions of CNTs with PEGylated dendrimers than with unPEGylated dendrimers [215]. The Panczyk group performed all-atom simulations of PEGylated and folic acid-functionalized CNTs that encapsulate doxorubicin, showing the release of doxorubicin from CNTs at acidic pH but not at neutral pH [216]. Fullerene molecules were also included to the inner cavity of CNT functionalized with PEG and folic acid, where fullerenes act as magnetic pistons at acidic pH, leading to an increase in the release of doxorubicin from nanotube [217], which helps explain the effect of PEG on the efficiency of drug release as well as suggests the use of fullerene, as presented in Figure 9. Meran et al. simulated CNTs coated with PEGylated pyrene and showed the adsorption of PEGylated pyrene onto the CNT surface via π-π stacking interactions, which does not significantly depend on PEG length and CNT size [218]. Saberinasab et al. performed quantum-mechanics (QM) calculations and all-atom MD simulations of a mixture of PEGylated CNTs and Temozolomide (anticancer drug), showing the adsorption of Temozolomide on PEGylated CNT because of strong hydrogen-bond interactions [219]. Moradnia et al. also performed QM calculations and all-atom MD simulations of a mixture of PEGylated CNTs and Gemcitabine (anticancer drug), showing the effect of PEG size on the hydrogen-bond interactions of Gemcitabine with PEGylated CNT and water [220]. Overall, simulation studies focused on the conformation of PEG and its effect on CNT dispersion until the mid-2010s, but have recently focused more closely on the effect of PEGylation on the efficiency of drug release and the binding affinity to drugs and proteins.

## 5. Conclusions

All-atom and CG MD simulations have revealed much useful information about the structure and dynamics of PEGylated drug transporters such as proteins, peptides, liposomes, dendrimers, and CNTs, which cannot be easily captured by experiments. In 1995–2000s, all-atom and CG PEG FFs have been developed and used for simulations of short linear PEG and their interactions with small molecules and solvents, while recent advances in computer power and simulation methods have allowed simulations of large complexes of PEGylated drug carries and their interactions with anticancer drugs, plasma proteins, lipid membranes, and receptors.

Although the molecular simulation has proven to be a powerful tool for the in silico design of PEGylated drugs for the past two decades, there are still problems that need to be considered for the future work. Firstly, biological complexes, reaction kinetics and mass transport conditions of experiments and simulations differ, which precludes any quantitative comparison between simulations and experiments. For instance, there are hundreds of plasma proteins that flow through the bloodstream, and hundreds of membrane proteins that control cellular behavior and interactions with drug carriers. Recently, MD simulations start to simulate the corona formation of various plasma proteins [221] and human cell membranes composed of 63 different lipid species [222], showing promising efforts in mimics of biological systems. Secondly, more accurate FFs need to be developed to predict the interactions between PEG and other molecules such as nucleotides and amino acids. As reviewed above, all-atom and CG PEG FFs have accurately predicted the conformation and physical properties of PEG in solvent, but the prediction of their interactions with other molecules need to be improved. Lastly, large complexes of PEGylated drugs interacting with other molecules should be considered. This can be done by multiscale simulations of the transition between all-atom and CG models [223], where the conformation and dynamics can be equilibrated by CG simulations, and then CG coordinates can be converted to all-atom models that offer insights into the atomic-level interactions such as electrostatic, hydrophobic, and hydrogen-bond interactions. To achieve this, simulation methodologies need to be developed for the transition from CG to an all-atom model that can be compatible with various biomolecules, polymers, surfactants, and solvents. 

Despite these limitations, MD simulations have successfully interpreted experimental observations at nearly the atomic scale and determined the optimal size, structure, and grafting density of PEG. Moreover, an increase in computational speed and methodology development (e.g., multiscale simulations of the transition between all-atom and CG models) will allow for more realistic simulations of larger biological systems, leading to a promising tool for the rational design of highly efficient PEGylated drug delivery systems.
